# Lactoferrin/sialic acid prevents adverse effects of intrauterine growth restriction on neurite length: investigations in an *in vitro* rabbit neurosphere model

**DOI:** 10.3389/fncel.2023.1116405

**Published:** 2023-04-26

**Authors:** Britta Anna Kühne, Lara Gutierrez-Vázquez, Estela Sánchez Lamelas, Laia Guardia-Escote, Laura Pla, Carla Loreiro, Eduard Gratacós, Marta Barenys, Miriam Illa

**Affiliations:** ^1^Grup de Recerca en Toxicologia (GRET) i INSA-UB, Departament de Farmacologia, Toxicologia i Química Terapèutica, Facultat de Farmàcia i Ciències de l'Alimentació, Universitat de Barcelona, Barcelona, Spain; ^2^BCNatal | Fetal Medicine Research Center (Hospital Clínic and Hospital Sant Joan de Déu), Universitat de Barcelona, Barcelona, Spain; ^3^Institut d’Investigacions Biomèdiques August Pi i Sunyer (IDIBAPS), Barcelona, Spain; ^4^Center for Biomedical Research on Rare Diseases (CIBER-ER), Barcelona, Spain; ^5^German Centre for the Protection of Laboratory Animals (Bf3R), German Federal Institute for Risk Assessment (BfR), Berlin, Germany; ^6^Institut de Recerca Sant Joan de Déu, Esplugues de Llobregat, Spain

**Keywords:** fetal growth restriction, neural progenitor cells, *in vitro* techniques, sialic acid, melatonin, DHA, neurite outgrowth, neuroprotective therapies

## Abstract

**Introduction:**

Intrauterine growth restriction (IUGR) is a well-known cause of impaired neurodevelopment during life. In this study, we aimed to characterize alterations in neuronal development underlying IUGR and discover strategies to ameliorate adverse neurodevelopment effects by using a recently established rabbit in vitro neurosphere culture.

**Methods:**

IUGR was surgically induced in pregnant rabbits by ligation of placental vessels in one uterine horn, while the contralateral horn remained unaffected for normal growth (control). At this time point, rabbits were randomly assigned to receive either no treatment, docosahexaenoic acid (DHA), melatonin (MEL), or lactoferrin (LF) until c-section. Neurospheres consisting of neural progenitor cells were obtained from control and IUGR pup’s whole brain and comparatively analyzed for the ability to differentiate into neurons, extend neurite length, and form dendritic branching or pre-synapses. We established for the very first time a protocol to cultivate control and IUGR rabbit neurospheres not only for 5 days but under long-term conditions up to 14 days under differentiation conditions. Additionally, an in vitro evaluation of these therapies was evaluated by exposing neurospheres from non-treated rabbits to DHA, MEL, and SA (sialic acid, which is the major lactoferrin compound) and by assessing the ability to differentiate neurons, extend neurite length, and form dendritic branching or pre-synapses.

**Results:**

We revealed that IUGR significantly increased the neurite length after 5 days of cultivation in vitro, a result in good agreement with previous in vivo findings in IUGR rabbits presenting more complex dendritic arborization of neurons in the frontal cortex. MEL, DHA, and SA decreased the IUGR-induced length of primary dendrites *in vitro*, however, only SA was able to reduce the total neurite length to control level in IUGR neurospheres. After prenatal *in vivo* administration of SAs parent compound LF with subsequent evaluation *in vitro*, LF was able to prevent abnormal neurite extension.

**Discussion:**

We established for the first time the maintenance of the rabbit neurosphere culture for 14 days under differentiation conditions with increasing complexity of neuronal length and branching up to pre-synaptic formation. From the therapies tested, LF or its major compound, SA, prevents abnormal neurite extension and was therefore identified as the most promising therapy against IUGR-induced changes in neuronal development.

## 1. Introduction

Intrauterine growth restriction (IUGR) is defined as a significant decrease in fetal growth rate resulting in a birth weight below the 10th percentile of the corresponding gestational age (Sharma et al., 2016). The prevalence accounts for 5–10% of all pregnancies, and amounts to approximately 600,000 cases in Europe, being therefore a serious health problem (Kady and Gardosi, 2004). Placental insufficiency, the main cause of IUGR, chronically decreases the blood flow and nutrient supply to the developing fetus resulting in an unfavorable *in utero* environment with chronic hypoxia conditions. This situation results to a wide range of abnormal trajectories of brain development including grey (GM) and white matter (WM) injury (Esteban et al., 2010; Pla et al., 2020), which are associated with short- and long-term neurodevelopmental damage and cognitive dysfunctions (Mwaniki et al., 2012; Batalle et al., 2014; Eixarch et al., 2016). This WM injury is tightly related to impaired oligodendrocyte development and myelination (Tolcos et al., 2011; Eixarch et al., 2012; Reid et al., 2012; Rideau Batista Novais et al., 2016), while GM impairment is, in this case, related to altered neuronal connectivity, as described in humans (Batalle et al., 2012) including irregular neurite and dendritic processes in cerebellar cortex and hippocampus, as discovered in sheep, guinea pig and rabbit models of IUGR (Dieni and Rees, 2003; Piorkowska et al., 2014; Pla et al., 2020). Previous investigations of our group unraveled IUGR induced impaired oligodendrogenesis in an *in vitro* rabbit neurospheres model, which correlates very well with clinical outcomes of WM injury (Kühne et al., 2022). In the current study, we have focused on investigating neuronal development using the same model but studying differentiation of neurons, their neurite outgrowth followed by dendritic branching and network formation *in vitro*. Besides that, we also used the model to test the potential neuroprotective therapies docosahexaenoic acid (DHA), melatonin (MEL), lactoferrin (LF), and LF’s main metabolite sialic acid (SA).

Currently, there is no efficient neuroprotective treatment to avoid deleterious consequences of IUGR in brain development (Lees et al., 2022). Several clinical and experimental assessments give evidence that early postnatal approaches like breastfeeding (Rao et al., 2007), individualized newborn developmental care and assessment program (Als et al., 2012), and environmental enrichment (Illa et al., 2018) can partially ameliorate the neurodevelopmental impairment caused by IUGR. However, all these strategies have been applied after birth, at a time point when adverse effects of IUGR on brain development have already occurred. The application of a treatment during the prenatal period, a “critical window of opportunity” (Andersen, 2003) is a unique chance to complement postnatal approaches which should not be missed. But to discover efficacious prenatal neuroprotective treatments, it is essential to first deepen the understanding of the mechanisms causing neurostructural changes underlying fetal programming due to IUGR, and for that, to have a good experimental model is indispensable.

**Figure 1:**
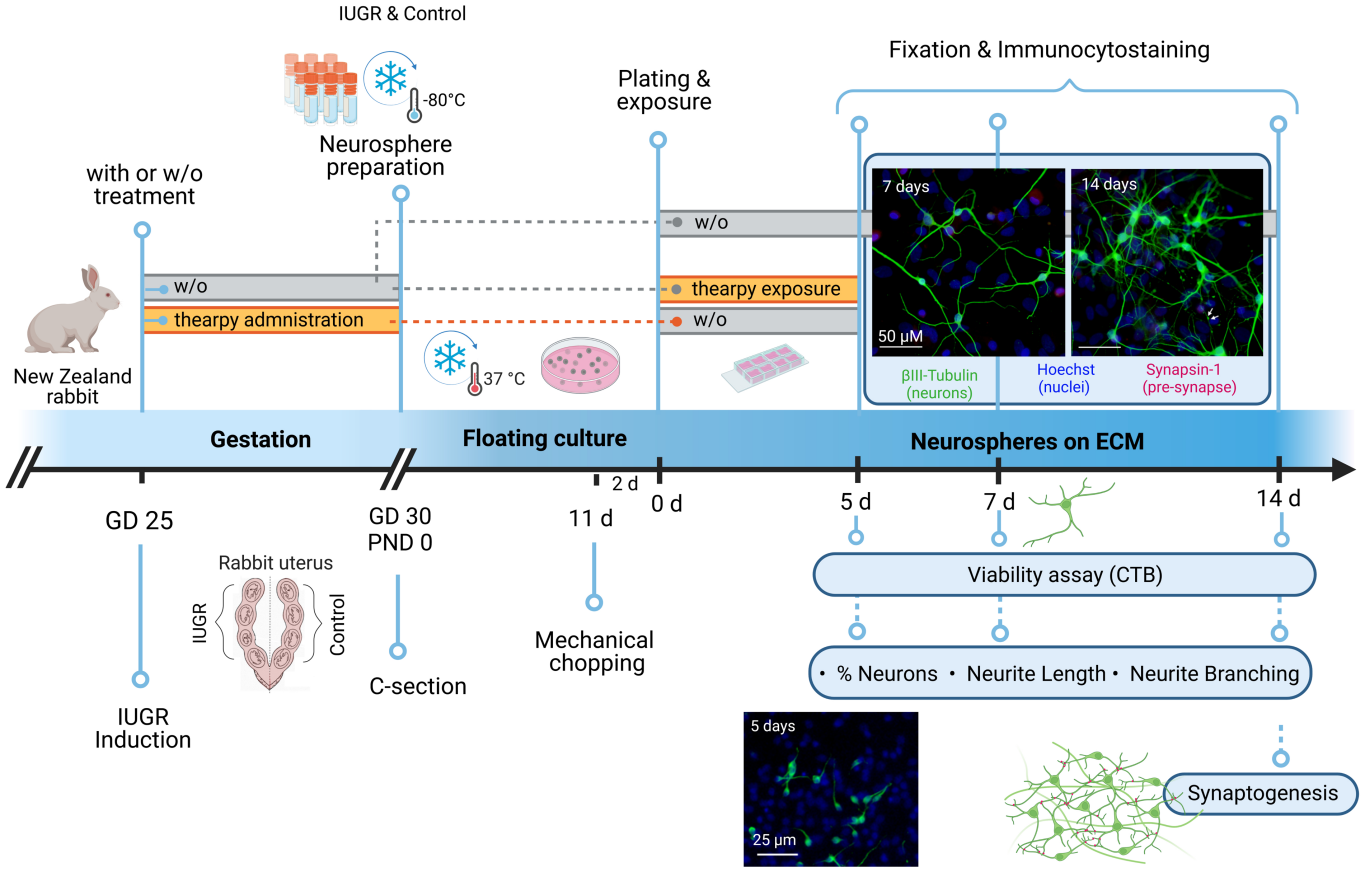
Experimental setup. At gestational day 25, IUGR was induced in one uterine horn of pregnant New Zealand rabbits, while the contralateral horn remained as control. Rabbits were kept until C-section on GD30 with or without (w/o) administration of therapies. Neurospheres were obtained from control and IUGR rabbit pup’s whole brain on PND0 and stored at −80°C. neurospheres were cultivated in a floating culture until mechanical chopping after 11 days. After 2 days neurospheres were plated on an 8-chamber slide previously coated with PDL/Laminin with or w/o exposure to therapies under differentiation conditions. The following endpoints were analyzed after 5, 7, or 14 days: Viability, % neurons, neurite length, and neurite branching (determined by the number of dendritic branching per neuron). Pre-synaptic formation was determined by the % of synapsin-1+ neurons and analyzed after 14 days. The effect of therapies on neurogenesis was assessed after 5 days under differentiation conditions. Rectangle bars = time of administration or exposure, blue circles = endpoints. w/o = without, GD = gestational day, PND = postnatal day, c-section = cesarean section, ECM = extracellular matrix, CTB = cell titer blue. Created with BioRender.com with the license number PY255AV1EQ.

Eixarch et al. (2009) developed an experimental IUGR model in pregnant rabbits mimicking placental insufficiency leading to neurodevelopmental symptoms of IUGR which highly correlate with clinical outcomes including postnatal functional and structural discrepancies (Eixarch et al., 2009, 2012). Previous studies using this animal model discovered neonatal as well as long-term persistence of brain reorganization and changes in cerebral network organization induced by IUGR (Eixarch et al., 2012; Illa et al., 2013; Batalle et al., 2014). These results agreed with clinical investigations that 1 year old infants who suffered IUGR also present alterations in structural brain connectivity based on connectomics studies (Batalle et al., 2012). The species rabbit was selected due to its higher similarity to human neurodevelopment compared to other species according to a precocial score established by Workman et al. (2013). Rabbits resemble humans in terms of circulatory changes during gestation, placentation, and brain maturation which occurs primarily postnatally in both species (Derrick et al., 2004; Carter, 2007; Workman et al., 2013). Combining clinical findings with the neurological changes observed in rabbits indicates that the rabbit IUGR model is a suitable model to assess IUGR-induced alterations in humans (Bassan et al., 2000; Eixarch et al., 2009, 2011; Barenys et al., 2021). To understand better, which basic cellular processes are altered during brain development under IUGR, our group established an *in vitro* model based on primary rabbit neuronal progenitor cells (NPCs) (Barenys et al., 2021). In this model, rabbit NPCs obtained from control and IUGR pups are cultured as three-dimensional (3D) cell aggregates known as neurospheres. Neurospheres are able to imitate basic courses of brain development such as NPC proliferation, migration and differentiation into the brain effector cells neurons, oligodendrocytes and astrocytes (Moors et al., 2007, 2009; Breier et al., 2010; Gassmann et al., 2010; Schreiber et al., 2010; Barenys et al., 2017). Because of its 3D structure encompassing multiple cell types, the neurosphere model is a valuable test system for studying a wide range of neurodevelopmental processes guided by a broad variety of cellular pathways (Gassmann et al., 2010, 2014; Baumann et al., 2016; Barenys et al., 2017, 2021; Dach et al., 2017; Masjosthusmann et al., 2019). In a cost-efficient and animal-reduction approach, with this model we were able to test a much wider concentration range of potential therapies *in vitro* compared to classical *in vivo* experiments. After that, and to confirm the findings, on the day of IUGR induction, potential therapies were administered to pregnant animals *in vivo* to subsequently investigate their prenatal effects in the neurosphere model. A detailed description of the experimental setup is displayed in Figure 1.

By using the rabbit neurosphere assay, our group revealed previously IUGR-induced adversity on the differentiation rate of pre-myelinating oligodendrocytes and discovered two therapies, DHA and MEL, reverting the reduced oligodendrocyte differentiation after *in vitro* exposure and preventing it after *in vivo* administration to the pregnant rabbit (Kühne et al., 2022). This previous study demonstrated that the novel rabbit *in vitro* neurosphere assay is able to accurately predict the *in vivo* outcome regarding oligodendrocyte differentiation (Kühne et al., 2022). DHA is also described to facilitate myelin formation, neurotransmitter synthesis, and sustaining synaptogenesis and neuronal network (Greenberg et al., 2008; Gil-Sánchez et al., 2010; Lauritzen et al., 2016), while MEL is depicted to reduce fetoplacental oxidative stress and white-and grey-matter damage in a sheep model of placental insufficiency (Rees et al., 2011; Miller et al., 2014). LF, a SA-rich glycoprotein, was considered as a promising candidate as it enhances neuronal growth, synaptic connectivity as well as placental development (Lopez et al., 2008; Wang, 2016). We selected MEL, DHA and LF due to their promising potential to improve impaired neurogenesis. Due to very limited solubility of LF in the cell culture medium, we used its main metabolite SA for the *in vitro* experiments. Further, we assessed their safety and efficacy on several neuronal endpoints: Neuron differentiation, neurite length, number of dendrites per neuron, as well as cell viability.

## 2. Materials and methods

### 2.1. IUGR induction

All animal experimentation procedures were approved by the Ethics Committee for Animal Experimentation (CEEA) of the University of Barcelona. All protocols were accepted by the Department of Environment and Housing of the Generalitat de Catalunya with the license number 11126, date of approval 24/5/2021, and the procedure CEEA number OB 340/19 SJD. The method of IUGR induction was previously described in Eixarch et al. (2009). Briefly, IUGR was induced at the 25th gestational day (GD) in pregnant New Zealand rabbits by surgical ligature of 40–50% of the uteroplacental vessels of each gestational sac of one uterine horn, while the contralateral horn was left for normal growth. Caesarean section was carried out at GD30 to obtain IUGR and control pups.

### 2.2. Administration of therapies *in vivo*

On the day of IUGR induction pregnant rabbit mothers were assigned to 4 different groups: without (w/o) administration, or with administration of MEL, DHA, or LF. The therapies were daily administered to the pregnant rabbit by releasing the solution with a syringe in the throat from the day of IUGR induction (GD 25) until the day of caesarean section (GD 30). Specific doses were determined as followed: MEL (10 mg /kg bw/day), DHA (37 mg/kg bw/day) and Lf (166 mg/kg bw/day). We refer to Kühne et al. (2022) for a detailed description about selection of *in vivo* doses, calculations and suppliers. For all treatment groups, the inclusion criteria for postnatal day 0 (PND0) IUGR pups was a birth weight lower and for control pups higher than the 25th percentile (39.7 g, Barenys et al., 2021). The number and birth weight of PND0 rabbit pups from each group was equal to the animals described in Kühne et al. (2022). Briefly 12 control and 10 IUGR pups from the group w/o were included from 8 rabbit mothers, 2 control and 2 IUGR rabbit pups were included from two different rabbit mothers for each treatment group.

From one rabbit pup’s whole brain, at least four independent experiments were performed.

### 2.3. Neurosphere preparation

The *in vitro* neurosphere culture was generated directly after decapitation at PND0, as described in Pla et al. (2022). Briefly, neural progenitor cells (NPCs) were isolated from rabbits’ whole brains by dissection, mechanical dissociation, digestion (20 min incubation with 20 U/ml papain [Worthington #LS003124] at 37°C), mechanical homogenization into a cell suspension, and centrifugation (10 min at 1200 rpm). The cell pellet obtained was resuspended in 1 ml of freezing medium (1:1; volume of pellet: volume of freezing medium [consisting in 70% (v/v) proliferation medium, 20% (v/v) fetal calf serum (FCS [Serva #1192002]), and 10% (v/v) DMSO]) and immediately stored at −80°C. Each cryo-vial was thawed by brief immersion in a 37°C water bath, and cells were transferred to 15 ml of proliferation medium preconditioned at 37°C and 5% CO_2_ for 2 h, and gentle resuspension. The cell suspension was centrifuged (10 min, 1,200 rpm), supernatant discarded and cells transferred to Poly-HEMA [Sigma #192066] coated dishes filled with proliferation medium [consisting in DMEM [Gibco #10569010] and Hams F12 [Gibco #31765027] 3:1 supplemented with 2% B27 [Gibco #17504044], and 20 ng/ml human recombinant epidermal growth factor (EGF [Gibco #PHG0313]) and recombinant human fibroblast growth factor 2 (FGF2 [R&D systems #233-FB]), Penicillin–Streptomycin (10,000 U/ml) [Gibco #15140122] supplemented with Rho kinase (ROCK) inhibitor Y-276322 [Tocris #1254] at a final concentration of 10 μM. Half of the volume of proliferation medium per petri dish was exchanged every 2–3 days by proliferation medium without ROCK inhibitor.

### 2.4. Neurosphere plating

IUGR and control brains derived neurospheres formed for 11 days in proliferation medium were always cultured in parallel. Two days before starting experiments, proliferating neurospheres were mechanically chopped to a size of 0.2 mm (McIlwain tissue chopper) to ensure homogeneous neurosphere size and spherical shape. Neurospheres were not chopped more than once. On the experiment plating day, 0.3 mm diameter neurospheres were selected and transferred in 8-chamber slides (Falcon #354118) previously coated with laminin [Sigma #L2020] and poly-D-lysin (PDL [Sigma #P0899]) containing differentiation medium [consisting in DMEM and Hams F12 3:1 supplemented with N2 [Gibco #17502048], Penicillin–Streptomycin (10,000 U/ml)]. The medium of 7-, and 14-day experiments were supplemented with 1% FCS. Half of the medium was renewed every 2–3 days. NPCs plated on a laminin/PDL coated surface radially migrated out of the sphere core and differentiated into effector cells. Each chamber contained five (5-day experiment) or six (7-and 14-day experiment) neurospheres representing replicates within one experiment, and at least three independent experiments were performed for every endpoint and exposure condition.

### 2.5. Therapy exposure *in vitro*

Compounds for neuroprotective therapy testing were dissolved in their corresponding vehicle depending on their maximum solubility (Table 1) and subsequently in differentiation medium. The effect of the potential therapies was assessed after 5 days of differentiation.

**Table 1:** Therapy exposure *in vitro*.

Therapy (synonym)	CAS Number	Max. Solubility	Concentration *in vitro*	MTC
MEL	73-31-4	100 μM (in DMSO)	0.1 – 0.3 – 1 – 3 μM	3 μM
DHA	6217-54-5	300 μM (in DMSO)	0.1 – 0.3 – 1 – 3 – 10 μM	10 μM
SA	131-48-6	30 μM (in DMSO)	0.1 – 0.3 – 1 – 3 – 10 μM	30 μM

Summary of the concentration range tested of potential therapies and their maximum solubility is indicated, as well as the maximum tolerated concentration (MTC) established in Kühne et al. (2022). The maximum solvent concentration was 0.1% (v/v) DMSO.

### 2.6. Immunocytochemistry

After 5, 7, or 14 days under differentiation conditions, neurospheres were fixed with paraformaldehyd (PFA) 4% for 30 min at 37°C, washed twice with PBS and stored in PBS until immunostained.

#### 2.6.1. Neuronal staining after 5 and 7 days

Neurospheres were incubated with a primary antibody solution containing 10% goat serum [Sigma #G9023] and 1:100 rabbit IgG anti-βIII-tubulin antibody [Sigma T2200] in PBS-T (PBS containing 0.1% Triton X-100) for 1 h at 37°C. After three washing steps with PBS, slides were incubated with secondary antibody solution containing 2% goat serum, 1:100 Hoechst 33258 [Sigma #B1166] and 1:200 Alexa 546 anti-rabbit IgG [Invitrogen #A-11030] in PBS for 30 min at 37°C.

#### 2.6.2. Co-staining of neurons and pre-synapses after 14 days

Neurospheres were incubated for 1 h at 37°C, with a primary antibody solution containing goat serum as blocking solution (10%), rabbit anti-βIII-tubulin IgG antibody (1:100), mouse anti-synapsin1 IgG antibody [synaptic system #106011] and PBS-T. After three washes with PBS, slides were incubated with secondary antibody solution containing 2% goat serum, 1:100 Hoechst 33258, 1:100 Alexa 488 anti-rabbit IgG [Invitrogen #A-11008], 1:100 Alexa 546 anti-mouse IgG and PBS for 30 min at 37°C.

After incubation with the respective secondary antibody solution and three washing steps with PBS, slides were mounted with Aqua Poly/Mount (Polyscience #18606) and stored at 4°C until image acquisition.

### 2.7. Image acquisition and analysis of neuronal endpoints

The endpoints “% of neurons,” “number of dendrites per neuron,” and “neurite length” were analyzed after 5, 7, and 14 days of differentiation, whereas “pre-synaptic formation” was only assessed after at time point 14 days (Figure 1). EGF [20 ng/ml] was used as a positive control for neuronal differentiation. Neurospheres were fixed, immunocytostained, and image analysis was carried out by taking two images of each migration area with a BX61 microscope (Olympus, Japan) with “UPlanFl 10x/0.30 Ph1” objective lens and using the ImageJ/Fiji 1.53q software. The number of nuclei (Hoechst staining) representing the total amount of cells was automatically counted using ImageJ/Fiji 1.53q software and neurons (βIII-tubulin+ cells) were manually counted. To determine the % of neurons the number of neurons was normalized to the number of nuclei.

The number of dendrites/neuron and their distances from the soma (neurite length) were manually measured by using ImageJ 1.53q after 5 days of differentiation. After 7 and 14 days, the number of dendrites/neuron and neurite length were assessed with the “Sholl analysis” of the ImageJ/Fiji 1.53q blinded to the experimental groups (L.G.V. and B.A.K.). The tool “Sholl analysis” was used to trace manually the different paths of dendrites calculating dendritic branching and length, as described in detail in Pla et al. (2020). The 8-bit tracing was constructed by using the Fiji plugins “Segmentation” and “Simple Neurite Tracer” as described in Binley et al. (2014). The total amount of dendrites was classified in primary, secondary, or tertiary dendrites depending on their point of division. The primary dendrites are born from the soma, the secondary ones from the primaries and so on. After 14 days the ability to generate pre-synapses was analyzed. Pre-synaptic puncta were defined as Synapsin-1 (pre-synaptic marker) co-localized with βIII-Tubulin+ cells. Pre-synaptic formation (synapsin-1+ neurons [%]) was determined by the number of neurons with synapsin-1+ puncta normalized by the total number of neurons. Analysis was evaluated in 5–6 neurospheres/condition, minimum 10 neurons/neurosphere in at least 3 independent experiments.

### 2.8. Cell viability

The cell viability was assessed with the CellTiter-Blue® cell viability assay (Promega #G8081). This assay is based on the measurement of mitochondrial reductase activity of living cells by conversion of resazurin to the fluorescent product resorufin. After 2 h of incubation with the reagent (1:3), medium was placed in a 96-well plate and read with FLUOstar Optima microplate reader. Neurospheres exposed to 10% DMSO (2 h) were used as lysis control.

### 2.9. Statistics

Statistical analysis was performed using GraphPad Prism v9. The difference between two samples was calculated with a two-tailed paired student’s *t*-test. Concentration-dependent effects were analyzed using one-way ANOVA. Time-course experiments including the comparisons of more than two groups were assessed by performing a two-way ANOVA. One-way and two-way ANOVA analysis was always followed by post-hoc test Bonferroni’s multiple comparison test. The respective statistical analysis is mentioned in each figure legend. The significance threshold was established at *p ≤ 0.05.

## 3. Results

### 3.1. IUGR increases neurite length after 5 days *in vitro*

**Figure 2:**
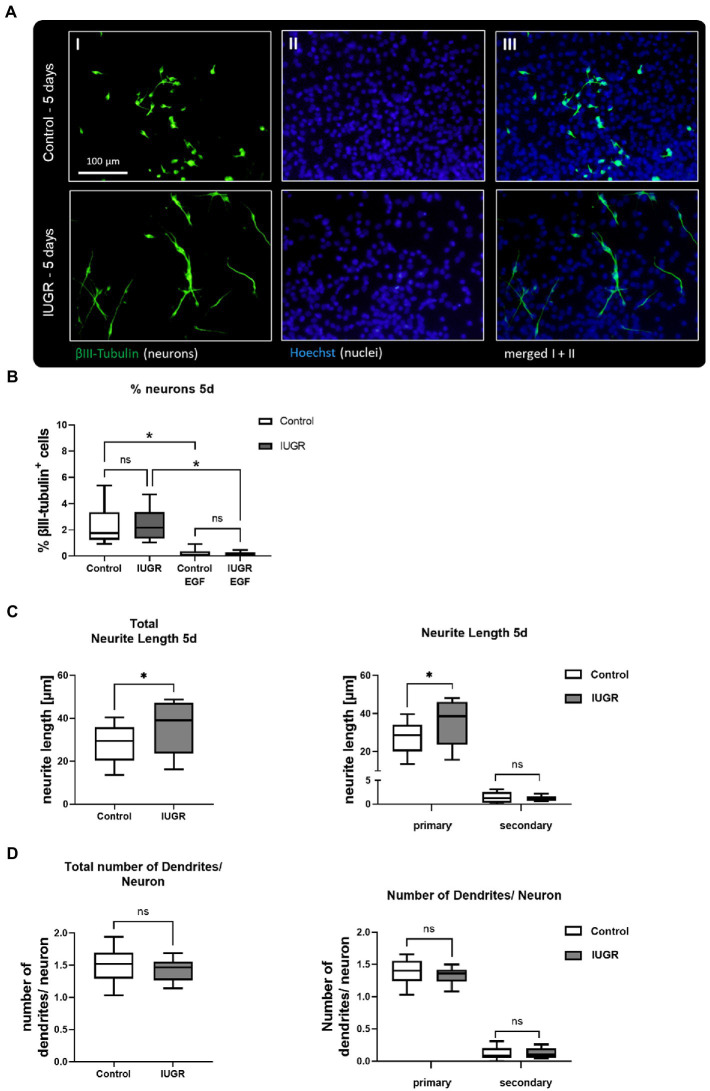
Neuronal development after 5 days after 5 days. **(A)** Representative pictures of neuronal marker βIII-Tubulin (I, green), nuclei marker Hoechst 33258 (II, blue) and merged (III) in control and IUGR neurospheres after 5 days under differentiation conditions. Control and IUGR neurospheres were tested for **(B)** % neurons, including the positive control EGF [20 ng/ml], **(C)** neurite length and **(D)** number of dendrites/neurons. Mean ± SEM; *p ≤ 0.05, ns: not significant. Comparison between two groups was analyzed by two-tailed paired *t*-test. Comparisons of more than two groups were assessed by performing a one-way ANOVA followed by Bonferroni’s multiple comparison test.

Neurospheres were prepared from 12 control and 10 IUGR rabbit pups with a significantly reduced body weight in the IUGR group as described in Kühne et al. (2022). Previous results from our group testing the impact of IUGR in the rabbit neurosphere model after 3 days in culture determined no difference between control and IUGR on the endpoint “% neurons” (Barenys et al., 2021). In the current study, we investigated the impact of IUGR on neuronal endpoints after 5 days *in vitro* to unravel if changes may occur at a later time point but we confirmed that the percentage of neurons was also not significantly different between control and IUGR at this time point (Figures 2A,B, Control: 2.25 ± 0.39% vs. IUGR: 2.34 ± 0.25%, *p* = 0.800). The positive control EGF significantly decreased the % of neurons in both groups (Figure 2B, Control: 0.21 ± 0.08%; IUGR: 0.15 ± 0.04%), and significantly increased the metabolic activity in comparison to the solvent control indicating a proliferative effect as expected for this growth factor (Supplementary Figure S1A) proving that the system is flexible and can react to external stimuli known to keep cells in a proliferating instead of in a differentiating status. The endpoint “neurite length” was measured by the distance from the soma to the neurite end (Figure 2C), and the “number of dendrites per neuron” revealed the degree of dendritic arborization (Figure 2D). After 5 days in culture, neurons of control, and IUGR neurospheres developed mainly primary dendrites and significantly fewer secondary dendrites per neuron (1.39 control primary vs. 0.12 control secondary dendrites/neuron, *p* < 0.0001; Figure 2D). Importantly, the total neurite length was significantly increased in IUGR neurospheres compared to the respective control value (Figure 2C, total control: 29.82 ± 2.84 vs. IUGR: 36.03 ± 3.46 μm, *p* = 0.011). This difference was due to the significantly larger primary neurites in IUGR neurospheres (Figure 2C, primary control: 28.50 ± 2.71 vs. IUGR: 34.81 ± 3.48 μm, *p* = 0.006), and not to differences in the secondary neurite length (Figure 2C, secondary control: 1.36 ± 0.35 vs. IUGR: 1.19 ± 0.16 μm, *p* = 0.943). The number of dendrites per neuron did not vary between control and IUGR after 5 days, neither the total number nor the number of primary or secondary dendrites (Figure 2D, total control: 1.51 ± 0.09 vs. IUGR: 1.44 ± 0.05 dendrites/neuron, *p* = 0.353).

### 3.2. IUGR increases the % of neurons after 14 days in a time-dependent manner

**Figure 3:**
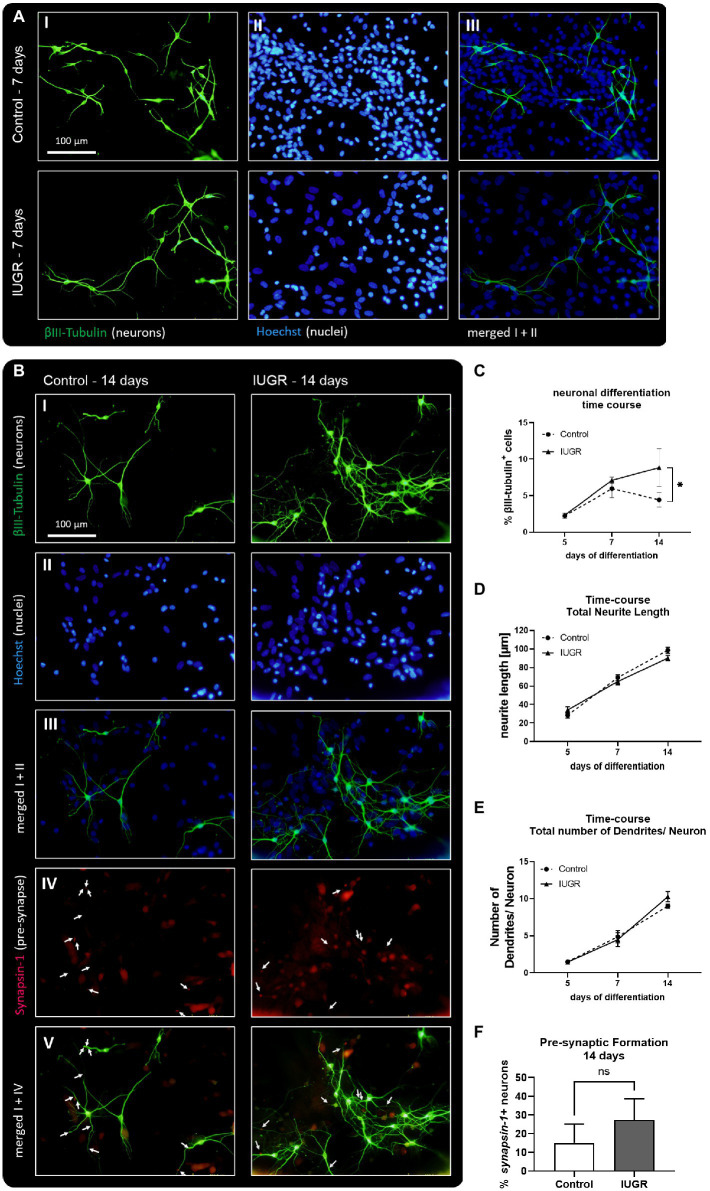
Neuronal development and network formation after 5, 7, and 14 days. **(A)** 7 days of neuronal differentiation in control and IUGR neurospheres. Representative pictures of neuronal marker βIII-Tubulin (I, green), nuclei marker Hoechst 33258 (II, blue) and merged (III). **(B)** 14 days of neuronal differentiation control and IUGR neurospheres. Representative pictures of (I) Neuronal marker βIII-Tubulin (green), (II) nuclei marker Hoechst 33258 (blue), (III) merged picture of neuronal and nuclei staining, (IV) pre-synaptic marker Synapsin-1, (V) merged picture of neuronal and synaptic staining. Scale bar = 100 μm. **(C)** Time course of neuronal differentiation from 3 to 14 days of differentiation [% βIII-Tubulin positive cells], **(D)** Time course of total neurite length/ neuron from 5–14 days. **(E)** Time course of total number of dendrites/neuron from 5–14 days. **(F)** Pre-synaptic formation: Rabbit neurospheres obtained from control and IUGR pups were cultured for 14 days and comparatively tested for the ability to generate pre-synapses. Pre-synaptic formation was determined by the number of neurons with synapsin-1 positive puncta normalized by the total number of neurons (synapsin-1+ neurons [%]). Analysis was evaluated in 6 neurospheres/condition, minimum 10 neurons/neurosphere in 3 independent experiments. Mean ± SEM; *p ≤ 0.05, ns: not significant. Time-course analysis was performed using two-way ANOVA followed by Bonferroni’s multiple comparison. Comparison between two groups was analyzed by two-tailed paired *t*-test **(F)**.

We established for the very first time the maintenance of rabbit control and IUGR neurospheres under long-term differentiation conditions [maximum time in culture previously described was 5 days *in vitro* (Barenys et al., 2021)] and examined the changes in neuronal morphology or network formation over time (Figure 3). Representative pictures of neurospheres cultured for 7 and 14 days *in vitro* revealed that both groups (IUGR and control) continued developing βIII-tubulin+ cells. After 7 days both neurosphere groups, control and IUGR, did not develop the pre-synaptic marker synapsin-1 (Supplementary Figures S2), but after 14 days they presented pre-synaptic formation (Figures 3A,B). In a time-course experiment including the time points 5, 7, and 14 days, a significant interaction between time and cases (control and IUGR) was observed (*p* = 0.0143). The percentage of βIII-tubulin+ cells was only slightly increased in the IUGR group compared to the control after 7 days. But remarkably, after 14 days, the percentage of neurons in IUGR neurospheres exceeded to a significant extent the percentage of neurons in the control group (Figure 3C, Control: 4.43 ± 0.98% vs. IUGR: 8.82 ± 2.61%, *p* = 0.005). By using this time-course approach we discovered that the neuronal differentiation rate is significantly faster in IUGR compared to control (Figure 3C, difference between the slopes of control and IUGR: *p* = 0.013), without affecting the viability at any time point indicating a specific effect on neuronal development by excluding a cytotoxic effect (Supplementary Figures S1B,C). The total neurite length and number of dendrites per neuron increased over time in both groups, control and IUGR, demonstrating a more complex neuronal morphology with extended neurites and more branched dendrites from 5 to 14 days (Figures 3D,E). After 14 days, the percentage of neurons developing pre-synaptic puncta (synapsin-1+ neurons) was measured (Figure 3B IV–V arrows, Figure 3F). However, no significant difference in the presynaptic formation between control and IUGR was discovered (Figure 3F, Control: 15.23 ± 9.97% vs. IUGR: 27.45 ± 11.36%, *p* = 0.277).

**Figure 4:**
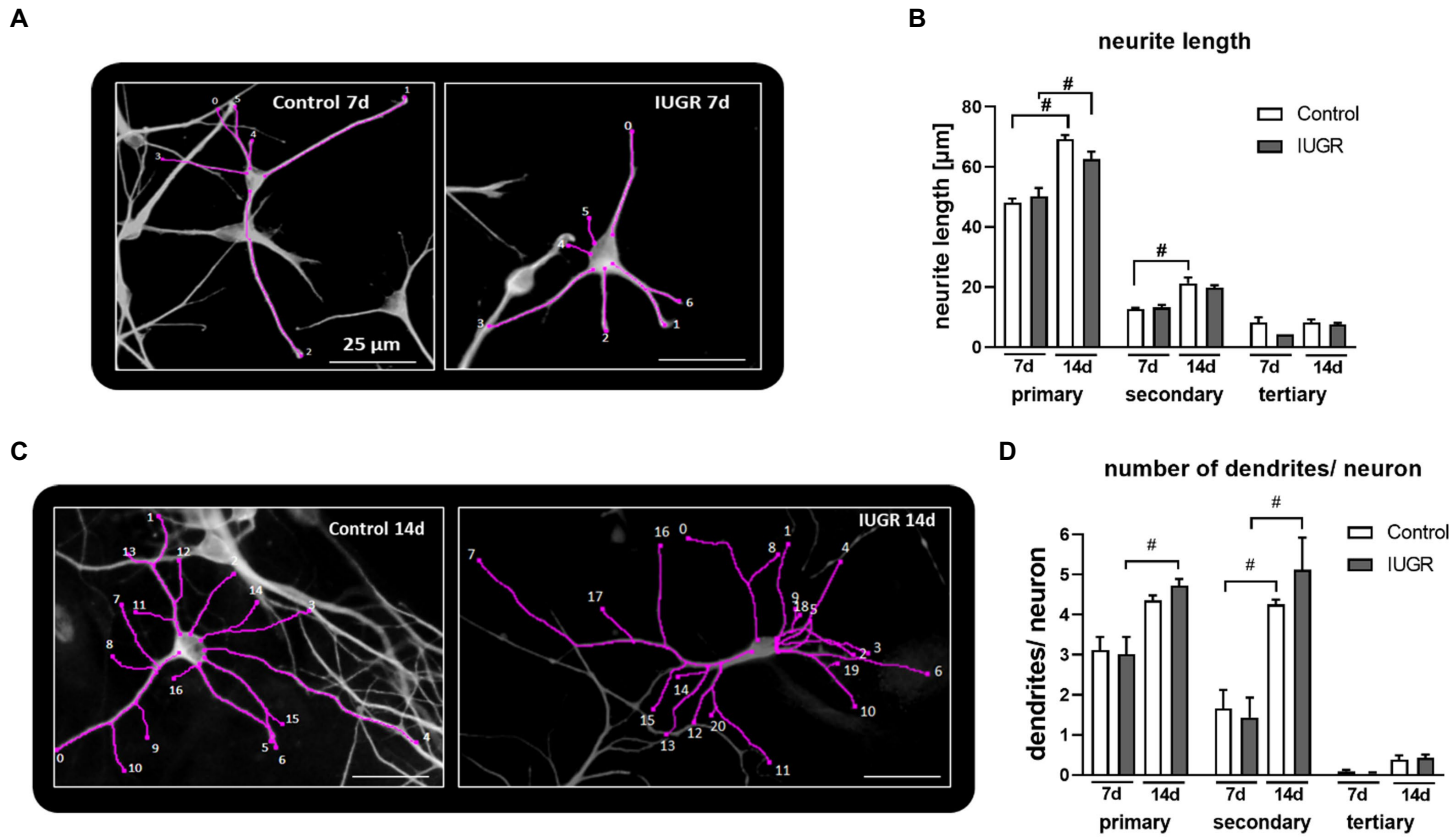
Length and number of primary, secondary and tertiary dendrites per neuron after 7 and 14 days. Example of “Sholl analysis” of a control (left) and IUGR (right) neuron, respectively, with traced and counted dendrites, after **(A)** 7 and **(B)** 14 days of differentiation, scale bar = 25 μm. Control and IUGR neurospheres were cultured for 7 or 14 days and comparatively tested for the **(C)** neurite length of primary, secondary and tertiary dendrites, **(D)** number of primary, secondary and tertiary dendrites/neuron. Analysis was evaluated in 6 neurospheres/condition, minimum 10 neurons/neurosphere in at least 3 independent experiments. Mean ± SEM; *p ≤ 0.05 control vs. IUGR. #: *p* ≤ 0.05 7 d vs. 14 d. Analysis was performed using two-way ANOVA followed by Bonferroni’s multiple comparison.

To investigate the development of neurite length and dendritic arborization in more detail, we measured not only their total number and length but also the number and length of primary, secondary, and tertiary dendrites after 7 and 14 days *in vitro* (Figure 4). While NPCs cultured for 5 days only formed neurons with primary and secondary dendrites (Figures 2C,D), NPCs cultured for 7 and 14 days established a more advanced neuronal phenotype including tertiary dendrites (Figure 4). Representative pictures display the measurement of neurite length and the number of primary, secondary, and tertiary dendrites of control and IUGR neurons after 7 and 14 days using the “Sholl analysis” (Figures 4A,C). Primary dendrites from neurons of both groups (control and IUGR) significantly extended their length over time from 7 to 14 days (Figure 4B), whereas only the IUGR group significantly increased the number of primary dendrites/neuron over time (Figure 4D). Secondary dendrites of neurons of both groups increased their number from 7 to 14 days, while only the control group significantly increased the length of secondary dendrites (Figures 4B,D). Tertiary dendrites from neurons of both groups did not significantly expand their length or number over time.

### 3.3. Assessment of potential therapies after 5 days *in vitro*

We selected the time point 5 days *in vitro* for further assessments of potential therapies, because our results at this time point correlate very well with the situation described *in vivo* in a previous study investigating structural brain changes in a rabbit model of IUGR (Pla et al., 2020). This study observed a more advanced dendritic morphology in the frontal cortex of IUGR compared to control animals (Pla et al., 2020).

**Figure 5:**
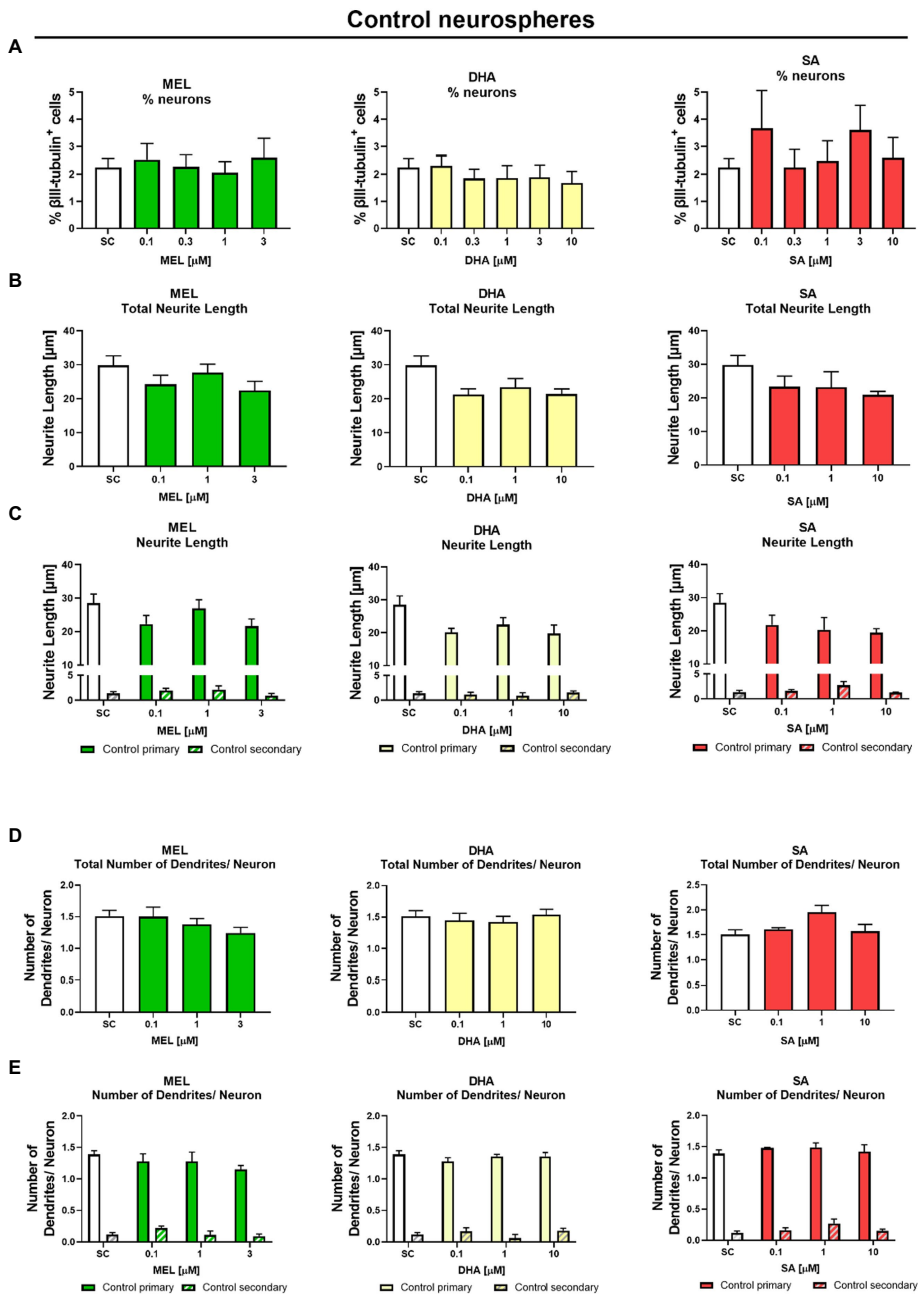
Exposure to potential therapies *in vitro* – safety evaluation after 5 days *in vitro*: Safety assessment of therapies on neuronal endpoints. Control neurospheres were tested for **(A)** % neurons [% βIII-tubulin+ cells], **(B)** total neurite length, **(C)** length of primary and secondary dendrites, **(D)** total number of dendrites/neuron, and **(E)** number of primary and secondary dendrites/neuron and exposed to increasing concentrations of Melatonin (MEL, green), DHA (yellow), or Sialic Acid (SA, red). Mean ± SEM. Concentration-dependent effects were analyzed using one-way ANOVA followed by Bonferroni’s multiple comparison test.

With the aim to revert adverse effects on neurogenesis induced by IUGR, we evaluated the safety and efficacy of 3 potential therapies MEL, DHA, and SA on the neuronal endpoints “% of neurons,” “neurite length,” and “number of dendrites per neuron,” as well as cell viability. In a previous study of our group using rabbit neurospheres, the maximum tolerated concentration (MTC) from MEL, DHA, and SA was determined in control neurospheres with the following criteria: viability was not lower than 70% of the solvent control (SC), migration distance and % of oligodendrocytes were not significantly reduced (Kühne et al., 2022). In the current approach, the additional criteria to set the MTC was “no significant adverse effect on any on the tested neuronal endpoints” in control neurospheres. Control neurospheres were exposed to potential therapies in a concentration-dependent manner for 5 days under differentiation conditions *in vitro* (Figure 5). None of the tested therapies adversely disturbed the tested endpoints (Figure 5, Supplementary Figure S3) and the MTC of each compound was set at the highest tested concentration for each compound, which was in accordance with the results in Kühne et al. (2022). A summary of these concentrations is presented in Table 1.

**Figure 6:**
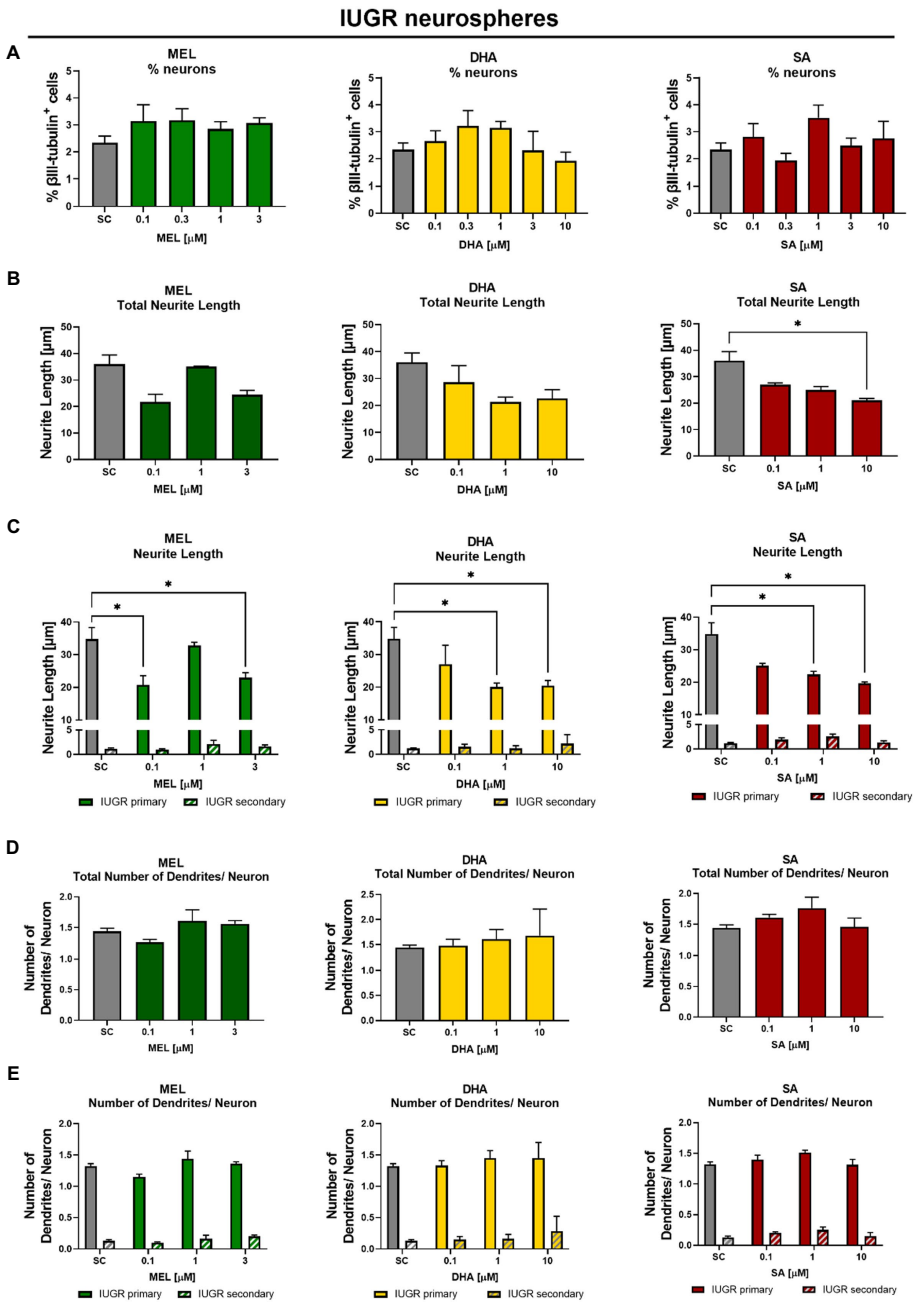
Exposure to potential therapies *in vitro* – efficacy evaluation after 5 days *in vitro*: Effect of therapies on neuronal endpoints. IUGR neurospheres were tested for **(A)** % neurons [% βIII-tubulin+ cells], **(B)** total neurite length, **(C)** length of primary and secondary dendrites, **(D)** total number of dendrites/neuron, and **(E)** number of primary and secondary dendrites/neuron and exposed to increasing concentrations of Melatonin (MEL, green), DHA (yellow), or Sialic Acid (SA, red). Mean ± SEM; *p ≤ 0.05 SC vs. treatment. Concentration-dependent effects were analyzed using one-way ANOVA followed by Bonferroni’s multiple comparison test. Comparison of more than two groups were assessed by performing a two-way ANOVA followed by Bonferroni’s multiple comparison test **(C,E)**.

The main interest was to find a concentration of the selected therapies which reverts the effects of IUGR. The cell viability determined by metabolic activity was always performed in the same experiments to distinguish between a specific effect and a general cytotoxic effect (Supplementary Figure S3). IUGR neurospheres were exposed to increasing concentrations of the selected therapies up to their MTC (Table 1). MEL, DHA, and SA did not significantly interfere with the % of neurons at any of the tested concentrations neither in control nor in IUGR neurospheres (Figures 5A, 6A). Our focus lied on decreasing the neurite length of the IUGR group because the total neurite length and the length of primary dendrites were significantly increased by IUGR after 5 days *in vitro.* MEL did not significantly reduce the total neurite length in IUGR neurospheres in none of the tested concentrations (Figure 6B). However, the lowest (0.1 μM) and the highest (3 μM) concentration of MEL significantly reduced the length of primary dendrites by presenting a stronger effect in the lowest concentration (Figure 6C, 0.1 μM MEL 20.70 ± 2.86 μm vs. SC 34.81 ± 3.48 μm, *p* = 0.004). MEL induced a non-monotonic response without showing a concentration-dependent effect or impact on the total neurite length, that is why MEL was not considered as the most favorable therapy against IUGR induced adverse effects on neurite length. Likewise, DHA did not present a significant reduction in the total neurite length at any of the tested concentrations (Figure 6B). Nevertheless, DHA showed a concentration-dependent effect on primary dendrites decreasing their length significantly (*p* = 0.001, Figure 6C). Even though DHA showed a positive effect on primary dendrites, the total length of neurites could not be improved. On the contrary, the exposure of SA to IUGR neurospheres prompted a concentration-dependent effect on total neurites and primary dendrites by significantly reducing their length. 10 μM SA significantly decreased the total neurite length (21.03 ± 0.75 μm vs. SC 36.03 ± 3.46 μm, *p* = 0.05), and 1 and 10 μM SA significantly reduced the length of primary dendrites (1 μM SA 22.43 ± 0.93 μm, *p* = 0.022; 10 μM SA 19.64 ± 0.5, *p* = 0.003). The total number of dendrites per neuron was not altered by any of the tested therapies (Figure 6D), neither the number of primary nor secondary dendrites per neuron (Figure 6E). Based on these findings, SA was selected to be the best candidate in reverting neurite extension induced by IUGR *in vitro*.

### 3.4. Administration of therapies *in vivo*–evaluation *in vitro*

To investigate the transferability of the *in vitro* results to the *in vivo* situation, we randomly assigned pregnant rabbits to different groups and administered MEL, DHA, or LF daily from the day of IUGR induction until C-section. SA is the main metabolite of LF, and therefore not SA but the parent compound LF was selected for the treatment *in vivo*. The body weight of the PND0-IUGR pups from all groups (with or without treatment; w/o) was significantly lower than the body weight of the respective control group, indicating that the treatments had no effect on the body weight [see Table 1 in Kühne et al. (2022)].

**Figure 7:**
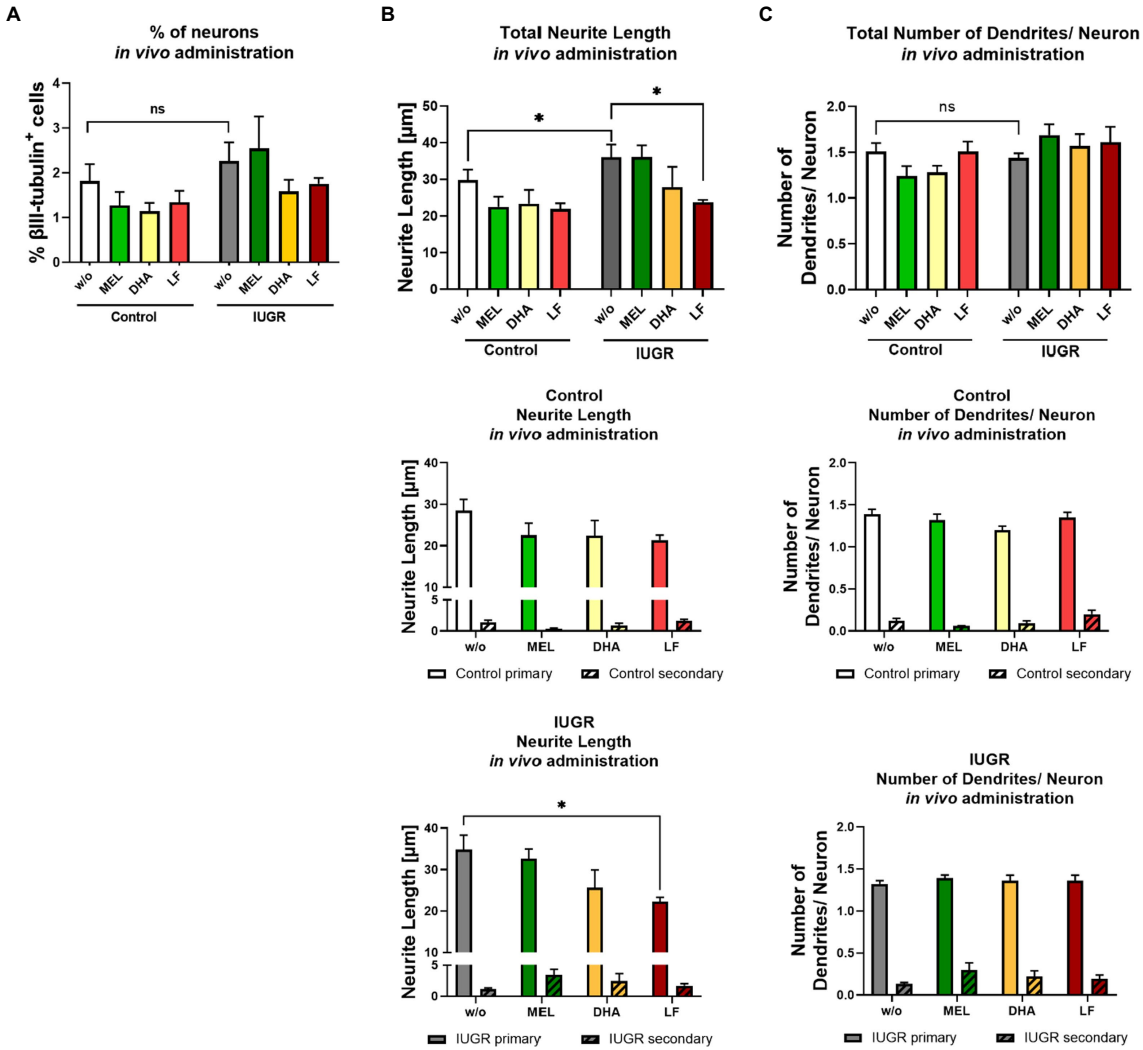
Administration of potential therapies *in vivo* – evaluation after 5 days *in vitro*. Pregnant rabbits received no treatment (w/o), MEL (10 mg/kg bw/day, green), DHA (37 mg/kg bw/day, yellow) or LF (166 mg/kg bw/day, red) at the day of IUGR induction until cesarean section. Neurospheres obtained from Control and IUGR pups were tested for **(A)** % neuronal differentiation (% βIII-tubulin+ cells), **(B)** total neurite length and below length of primary and secondary dendrites in control and IUGR neurospheres **(C)** total number of dendrites/ neuron and below number of primary and secondary dendrites in control and IUGR neurospheres; Mean ± SEM; *p ≤ 0.05, ns: not significant. Upper row: Data was analyzed using one-way ANOVA. Comparison of more than two groups (treatment, primary and secondary dendrites) was assessed by two-way ANOVA. Both followed by Bonferroni’s multiple comparison test.

Neurospheres were obtained from control and IUGR pup’s whole brain from the different treatment groups and analyzed for neuronal endpoints after 5 days under differentiation condition *in vitro*. The cell viability determined by metabolic activity was not significantly reduced compared to the control value in neurospheres of any treatment group (Supplementary Figure S4). The % of neurons was not significantly different between control and IUGR neurospheres obtained from pups from all treatment groups, which is in accordance with the effect observed *in vitro* (Figure 7A). Neurospheres obtained from IUGR pups prenatally administered to MEL did not display any improvement in neurite length, neither the total neurite length nor the length of primary or secondary dendrites (Figure 7B). Likewise, the prenatal administration of DHA to the pregnant rabbit could not prevent the adverse effect of IUGR on total neurite length, primary, or secondary dendrites (Figure 7B). However, neurospheres from IUGR pups of LF-treated rabbits presented a significant reduction in the total neurite length compared to the non-treated IUGR group (LF total: 23.71 ± 0.64 μm vs. w/o total: 36.03 ± 3.46 μm, *p* = 0.049, Figure 7B). The length of primary dendrites was also significantly reduced in IUGR neurospheres of the LF group compared to primary dendrites of the non-treated IUGR group (LF primary: 22.23 ± 1.10 μm vs. w/o primary: 34.81 ± 3.48 μm, *p* = 0.0045), while the length of secondary dendrites remained unaffected. In control neurospheres from the LF group, the neurite length of total and primary dendrites stayed on the level of w/o control neurospheres. Remarkably, these results are in good agreement with the results of the *in vitro* exposure to SA, the main metabolite of LF (Figures 6B,C). Finally, the total number of dendrites per neuron did not differ between any treatment group and w/o group (Figure 7C). None of the *in vivo* tested therapies influenced the number of primary or secondary dendrites per neuron, which was also in line with our *in vitro* results (Figures 6D,E).

Taking into account all results presented, the *in vitro* neurosphere assay correctly predicted the outcome of both, positive and negative results of the *in vivo* administration for the endpoints “% of neurons,” “total neurite length,” and “total number of dendrites” or “number of primary and secondary dendrites.” Merely the *in vitro* results of “length of primary and secondary dendrites” after exposure to MEL and DHA was not in accordance with the results of the prenatal *in vivo* treatment. Our findings revealed LF as the most promising therapy to prevent increased neurite length caused by IUGR.

## 4. Discussion

We used a previously established rabbit neurosphere model mimicking brain development under IUGR conditions (Barenys et al., 2021). The *in vitro* neurosphere system has been revealed to reproduce the clinical situation of WM injury by reducing the percentage of pre-oligodendrocytes in the IUGR group *in vitro* by accurately predicting the outcome *in vivo* with respect to oligodendrogenesis, which makes it a powerful and consistent tool to assess IUGR induced neurological alterations (Kühne et al., 2022). In this study, we assessed the impact of IUGR on neuronal development after 5, 7, and 14 days and pre-synaptic formation after 14 days *in vitro*, as well as the safety and efficacy of potential therapies *in vitro* and after *in vivo* administration.

IUGR neurospheres presented a significant increase in the total neurite length as well as in the length of primary dendrites after 5 days *in vitro*. These results correlate very well with a previous *in vivo* study of our group, where IUGR rabbit pups (PND1) presented a more complex branched morphology in frontal cortex neurons. In that study, tertiary, quaternary and quinary dendritic branches were significantly increased in the IUGR versus the control group (Pla et al., 2020). With the same *in vivo* rabbit model, diffusion tensor imaging parameters were assessed revealing reduced fractional anisotropy (FA) in several brain regions of WM and GM including the frontal cortex of IUGR animals (Eixarch et al., 2012). A decrease in cortical FA is associated with an increase in dendritic expansion and branching of neurons (McKinstry et al., 2002) and also correlates well with the increased neurite length present in our IUGR *in vitro* culture.

Although the exact mechanism leading to these changes in neurite branching *in vitro* and *in vivo* is not known yet, a recent study of our group provides evidence of a possible underlying molecular mechanism: IUGR neurospheres present a significant higher expression of the adhesion molecule integrin-β1 at gene and protein level (Kühne et al., 2022). Integrin-β1 interacts with the extracellular matrix (ECM) protein laminin allowing the migration of NPCs to a distal destination and the alignment of glial cell in brain and cerebellar cortex (Graus-Porta et al., 2001; Förster et al., 2002; Belvindrah et al., 2007; Barenys et al., 2017; Kühne et al., 2019). But integrin-β1 is not only involved in migration but also in the extension of neurite length and arborization in the developing brain as studies have shown that integrin-β1 deficiency in cells from partial knock-out mice were not able to evolve dendritic branching (Marrs et al., 2006; Belvindrah et al., 2007). Also, integrin-β1 blocking or inhibition experiments reduced or avoided neurite extension and branching on laminin (Moresco et al., 2005; Warren et al., 2012; Ortiz-Romero et al., 2018). In view of the finding that IUGR neurospheres overexpress integrin-β1, future experiments to prove if this is the underlying mechanism of the changes observed in neurites, such as plating control and IUGR neurospheres on an ECM with decreasing laminin concentrations to discover whether the extended neurite length decreases with declining laminin concentrations, are needed.

We successfully established for the very first time the maintenance of the rabbit neurosphere culture for 7 and 14 days under differentiation conditions. This is of importance for research groups working with the rabbit model and needing longer-term culture periods to model longer exposure to toxicants or to therapeutic agents. Over that time in culture, both control and IUGR neurospheres developed a more complex neuronal phenotype including more dendrites and longer neurites. This outcome demonstrates that our rabbit neurosphere system is able to reflect spatiotemporal processes of brain development with increasing complexity of the nervous system as described for human neurospheres (Kim et al., 2006).

In the time-course experiment, the percentage of neurons in IUGR significantly exceeded the % of neurons in control neurospheres after 14 days. This increase in percentage of neurons was not previously described in the *in vivo* IUGR model (it was not tested) and therefore needs further confirmation. For this reason, we decided not to focus on the prevention/reversion of this result for the evaluation of the therapies. After 14 days, both groups were able to develop the pre-synaptic marker synapsin-1, suggesting neuronal network formation. Our results indicate a faster network expansion in the rabbit compared to the human neurosphere culture. Neurons of primary human NPCs did not develop synaptic markers until 28 days under differentiation conditions and human induced pluripotent stem cells developed synaptic markers at first after 28 days in culture forming an electrically active neuronal network (Hofrichter et al., 2017; Nimtz et al., 2020). We did not find a difference in pre-synaptic marker formation between control and IUGR, but to our knowledge, IUGR induced disturbance of neuronal function including signal transmissions has never been studied yet in an *in vitro* model of IUGR. In future, it will be necessary to co-label pre-and post-synaptic compartments by including a post-synaptic marker such as PSD-95 to confirm the formation of synapses between neurons. However, it was challenging to maintain rabbit neurospheres for more than 7 days under differentiation conditions, and it was necessary to supplement the medium with FCS, which is known to improve viability but also induce neuronal differentiation in rabbit neurospheres (Barenys et al., 2021). If IUGR neurospheres need to be kept under restricted oxygen and nutrients supply *in vitro* for long-term culture to continue to be a correct model to study IUGR needs further clarification, thus we decided to focus on the differentiation day 5 in the current study.

To date no efficient treatment is available to improve adverse brain development occurring from IUGR, and neuroprotective therapies are urgently needed to be applied prenatally during the “critical window of opportunity” to protect or correct IUGR-induced brain damage (Andersen, 2003). Our rabbit model mimics the situation of late-onset IUGR, which is the most critical instance encompassing the highest incidence of IUGR cases with a very small time window between diagnosis and intervention (Figueras and Gratacos, 2017; Figueras et al., 2018). For the safety and efficacy testing of potential therapies MEL, DHA, and LF or its main metabolite SA we exposed the neurospheres for 5 days under differentiation conditions, as this time point presented several advantages: i) it is a short-time period in good agreement with the short intervention time described in late-onset IUGR cases in humans, ii) it is the time between IUGR induction *in vivo* and C-section, so the *in vitro* and *in vivo* treatments would be comparably long, and iii) the *in vitro* results on IUGR-induced changes in neurites highly correspond to previous *in vivo* findings (Pla et al., 2020). Regarding the safety of the selected therapies, none of the tested concentrations *in vitro* or after prenatal administration *in vivo* exhibited an adverse effect on viability or neuronal endpoints in control neurospheres indicating safe concentrations *in vitro* and *in vivo*. These results comply with the safety assessment of all tested compounds on the endpoint oligodendrogenesis as previously calculated in Kühne et al. (2022). In this earlier study, we identified MEL and DHA as the most promising therapies to prevent and revert IUGR adverse effects on oligodendrogenesis, while LF did not change impaired oligodendrocyte differentiation after IUGR induction (Kühne et al., 2022). The hormone MEL is a highly effective antioxidant, which readily crosses the placental and blood–brain barrier reducing fetoplacental oxidative stress and decreased white-and grey-matter damage in different models of IUGR (Rees et al., 2011; Miller et al., 2014; Castillo-Melendez et al., 2017). DHA is a long-chain omega-3 polyunsaturated fatty acid that is an important component of brain membrane phospholipids, which contributes to neuronal differentiation and signaling and accelerate myelination (Greenberg et al., 2008; Gil-Sánchez et al., 2010; Lauritzen et al., 2016). By exposure to different concentrations of MEL, DHA, and SA to IUGR neurospheres *in vitro*, all three tested compounds could revert the IUGR-induced length of primary dendrites, however, only SA was able to decrease the total neurite length in IUGR neurospheres. After *in vivo* administration only LF could prevent the neurite elongation in IUGR neurospheres and was therefore considered as the best candidate for the protection of neuronal development.

LF is an iron-binding, SA-rich glycoprotein known to act as anti-bacterial and anti-inflammatory compound protecting the development of brain and cognitive function (Wang, 2016). LF was reported to protect against immature brain injury by recovering cerebral GM and WM destruction in a rat model of hypoxia-ischemia after maternal supplementation with LF (van de Looij et al., 2014). SA is a monosaccharide that plays a key role in the synthesis of brain gangliosides and sialylated glycoproteins including polysialic acid (Poly-SA), which binds to the neural cell adhesion molecule (NCAM) and is crucial for the neurodevelopment (Weinhold et al., 2005; Bonfanti, 2006; Wang, 2016). (Burgess et al. (2007) discovered in an *in vitro* primary embryonic rat cell culture, that poly-SA limits the neurite elongation of septal neurons by preventing the interactions between integrin-β1 and laminin. The other way around, a removal of poly-SA enhances the laminin and integrin-β1 interaction, which was responsible for neurite outgrowth. Another group reported that a removal of poly-SA facilitated the development of immature neurons in adult mice (Coviello et al., 2021), thus showing the *in vivo* relevance of this interaction. These results are in line with our investigations revealing an overexpression of integrin-β1 in IUGR NPCs (Kühne et al., 2022) along with elongated neurite length in IUGR neurospheres, which could be reverted due to SA exposure *in vitro* and prevented by LF administration *in vivo*.

However, it is important to remark that we do not know if the increased neuronal arborization is harmful or is a mechanism compensating other adverse effects of IUGR. In previous *in vivo* experiments using the same rabbit model, IUGR animals did not present severe functional impairments at short-term (PND1) (Pla et al., 2020), but only at later time points (PND70) neurobehavioral and cognitive deficiencies could be evaluated and indeed memory and anxiety traits were detected in the IUGR animals (Illa et al., 2013, 2018). In future, we need studies evaluating long-term effects of LF in GM along with cognitive functional studies, which could not be assessed at the short-term evaluation in our animal species (Illa et al., 2023).

In summary, we established for the first time the sustainment of the rabbit neurosphere culture until 14 days under differentiation conditions with increasing complexity of neuronal length and branching up to pre-synaptic formation, mimicking spatiotemporal characteristics of brain development. IUGR neurospheres presented a significant increase in neurite length compared to control neurospheres after 5 days *in vitro*. The underlying mechanism of this effect in the IUGR group is not known, but we hypothesize that it could be attributed to an increase in the adhesion molecule integrin-β1, as previously discovered in Kühne et al. (2022). Supporting this hypothesis, SA was able to revert *in vitro*, and LF to prevent *in vivo* the induced neurite length in IUGR neurospheres. Whereas MEL and DHA could not improve the IUGR induced total neurite extension neither *in vitro* nor after prenatal treatment *in vivo*. For future applications in the clinical field it may be of high interest to develop a combined supplementation during pregnancy including DHA and/or MEL as protective agent against impaired oligodendrocyte differentiation (Kühne et al., 2022) and LF to prevent IUGR-induced alterations on neuronal development.

## Data availability statement

The original contributions presented in the study are included in the article/Supplementary material, further inquiries can be directed to the corresponding author.

## Ethics statement

The animal study was reviewed and approved by the Ethics Committee for Animal Experimentation (CEEA) of the University of Barcelona with the CEEA number OB 340/19 SJD and the Department of Environment and Housing of the Generalitat de Catalunya with the license number 11126. Date of approval 24/5/2021.

## Author contributions

BA, MB, and MI contributed to conception and design of the study. BA, LG-E, MB, and MI contributed to data analysis and interpretation of the data. BA, LG-V, ES, LP, and CL performed methodology, generated, and analysed data. EG contributed to resources and funding acquisition. MB and MI administrated and supervised the project. BA wrote the original draft of the manuscript. LG-E, MB, and MI critically revised and edited manuscript. All authors contributed to the article and approved the submitted version.

## Funding

This study has been funded by Instituto de Salud Carlos III, PI18/01763 (Co-funded by European Regional Development Fund. ERDF, a way to build Europe) and the ASISA Foundation. BK received a scholarship from Fundació Bosch i Gimpera (project number: 300155).

## Conflict of interest

The authors declare that the research was conducted in the absence of any commercial or financial relationships that could be construed as a potential conflict of interest.

## Publisher’s note

All claims expressed in this article are solely those of the authors and do not necessarily represent those of their affiliated organizations, or those of the publisher, the editors and the reviewers. Any product that may be evaluated in this article, or claim that may be made by its manufacturer, is not guaranteed or endorsed by the publisher.
