# Supplementary material for: Lactoferrin/sialic acid prevents adverse effects of intrauterine growth restriction on neurite length: investigations in an in vitro rabbit neurosphere model
Source: Front Cell Neurosci. 2023 Apr 26;17:1116405. doi: 10.3389/fncel.2023.1116405 (PMC10169722; doi:10.3389/fncel.2023.1116405)
Supplement: Supplementary file 1 [file Data_Sheet_1.docx]

Supplementary Material

Lactoferrin/Sialic acid prevents adverse effects of intrauterine growth restriction (IUGR) on neurite length: investigations in an in vitro rabbit neurosphere model

Running title: Lactoferrin prevents IUGR-induced neurite length

Britta Anna Kühne, Lara Gutiérrez Vázquez, Estela Sánchez Lamelas, Laia Guardia-Escote, Laura Pla, Carla Loreiro, Eduard Gratacós, Marta Barenys ^†^ and Miriam Illa *^, †^

† These authors contributed equally to this work and share last authorship

* **Correspondence:** Dr. Miriam Illa, [miriamil@clinic.cat](mailto:miriamil@clinic.cat)


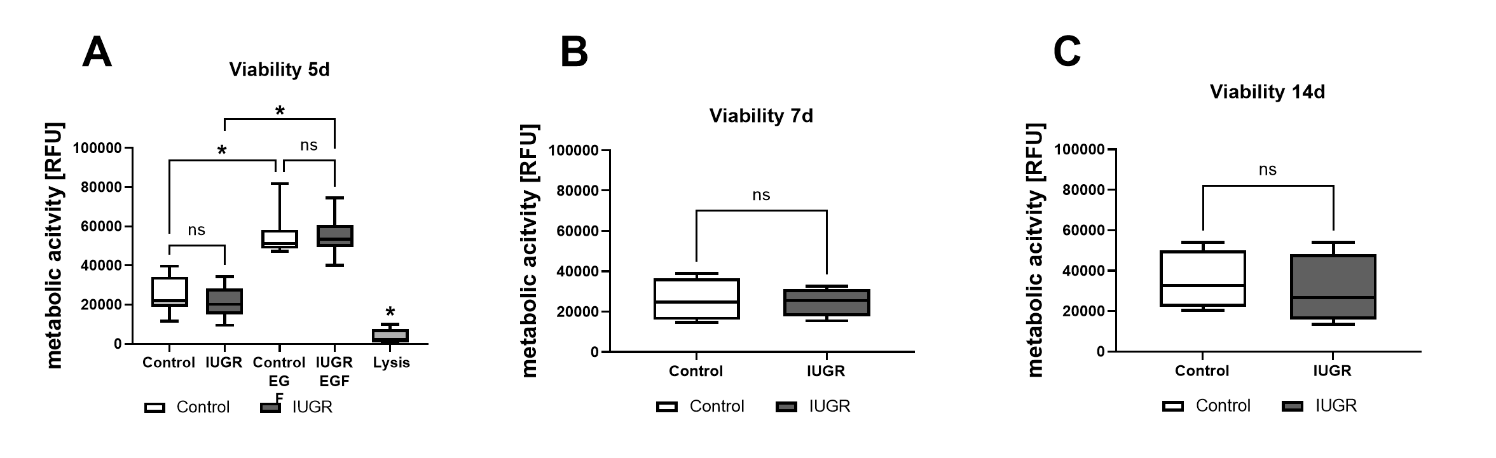


Supplementary Figure 1. Viability of control and IUGR neurospheres: 5, 7 and 14 days in vitro. Control and IUGR neurospheres were tested for viability determined by metabolic activity after (A) 5, (B) 7, and (C) 14 days under differentiation condition, including positive controls Lysis [10 % DMSO]. Mean ± SEM; * p≤0.05, ns: not significant. Comparison between two groups was analyzed by two-tailed paired t-test. Comparisons of more than two groups were assessed by performing a one-way ANOVA followed by Bonferroni’s multiple comparison test.


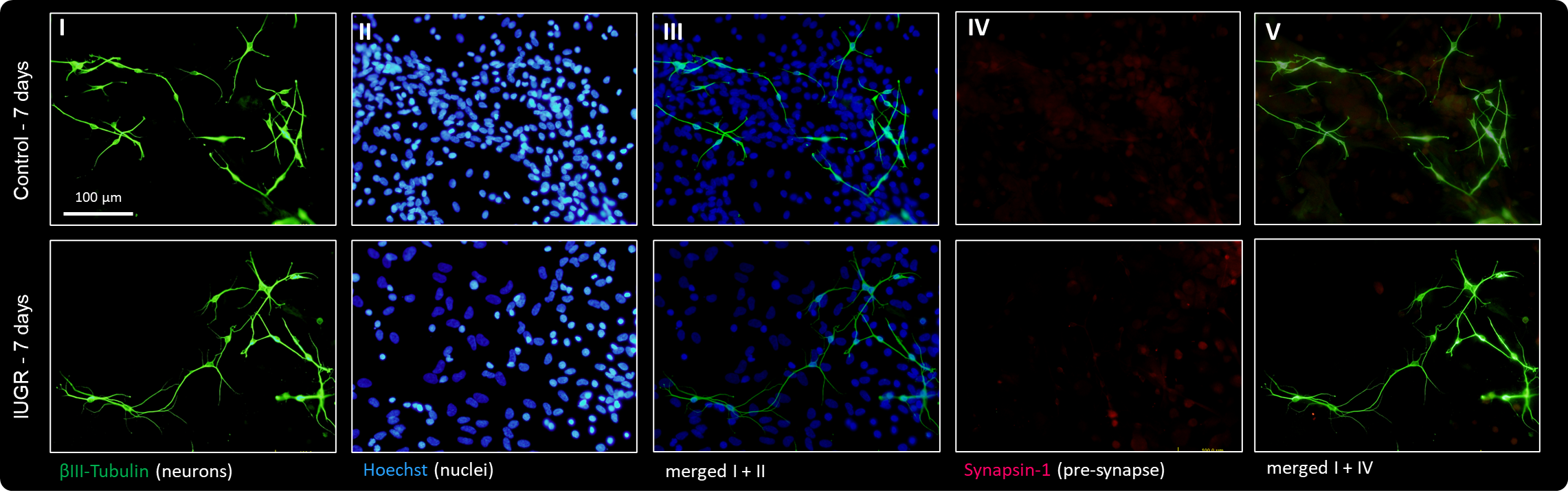


Supplementary Figure 2. Neuronal development 7 days. 7 days of neuronal differentiation in control and IUGR neurospheres. Representative pictures of (I) Neuronal marker βIII-Tubulin (green), (II) nuclei marker Hoechst 33258 (blue), (III) merged picture of neuronal and nuclei staining, (IV) pre-synaptic marker Synapsin-1, (V) merged picture of neuronal and synaptic staining. Scale bar = 100 µm.


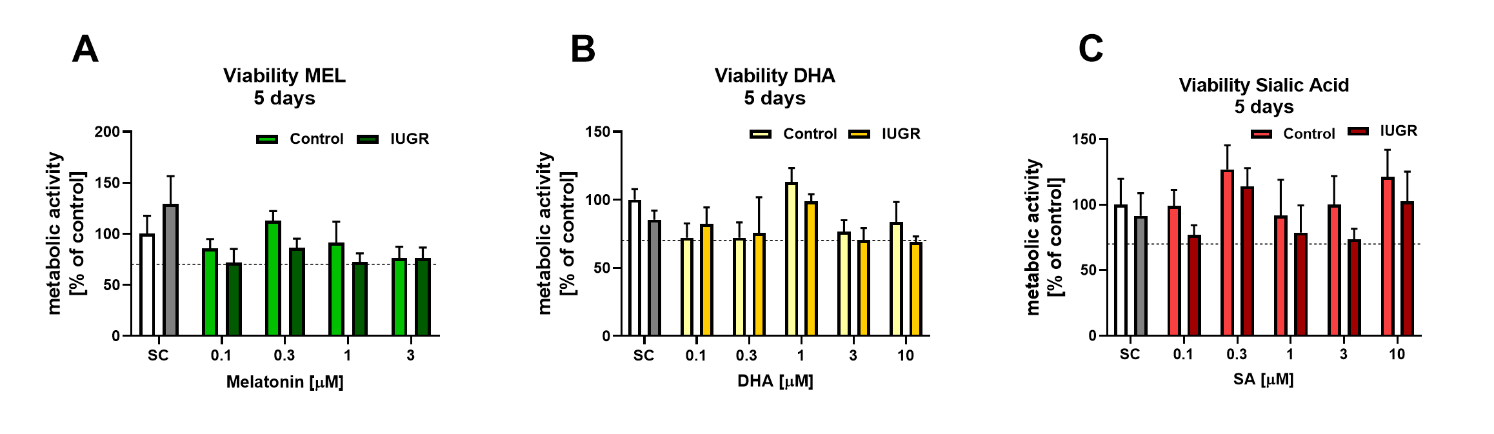


Supplementary Figure 3. Exposure to therapies *in vitro* – evaluation after 5 days *in vitro*: Safety assessment of potential therapies in viability. Control and IUGR neurospheres were tested for viability determined by metabolic activity after 5 days under differentiation condition and exposure to increasing concentrations of (A) Melatonin (MEL), (B) DHA, (C) Sialic Acid (SA). Dotted line: 70% metabolic activity. Mean ± SEM. Data was analyzed by two-way ANOVA followed by Bonferroni’s multiple comparison test.


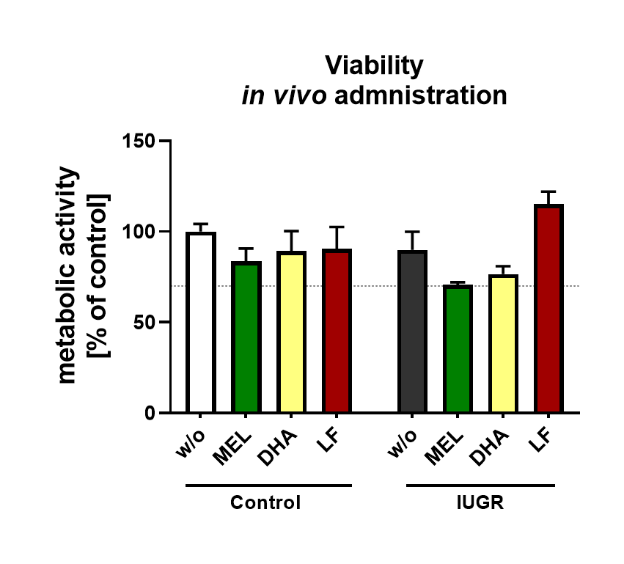


Supplementary Figure 4. Administration of therapies *in vivo* – evaluation after 5 days *in vitro*: Safety assessment of potential therapies in viability. Pregnant rabbits received no treatment (w/o) MEL (10 mg /kg bw/day, green), DHA (37 mg/kg bw/day, yellow) or LF (166 mg/kg bw/day, red) at the day of IUGR induction until C-section. Neurospheres obtained from Control and IUGR pups were tested for viability determined by metabolic activity; black dotted line: 70% metabolic activity. Mean ± SEM. Data was analyzed using one-way ANOVA, followed by Bonferroni’s multiple comparison test.
